# Expanding Access to Comprehensive Geriatric Evaluation via Telehealth: Development of Hybrid-Virtual Home Visits

**DOI:** 10.1007/s11606-023-08460-5

**Published:** 2024-01-16

**Authors:** Cathy C. Schubert, Lauren S. Penney, Ashley L. Schwartzkopf, Teresa M. Damush, Alaina Preddie, Soyna Flemming, Jennifer Myers, Laura J. Myers, Anthony J. Perkins, Ying Zhang, Dawn M. Bravata

**Affiliations:** 1 Geriatrics and Community Service, Richard L. Roudebush VA Medical Center, Indianapolis, IN USA; 2 Department of Internal Medicine, Indiana University School of Medicine, Indianapolis, IN USA; 3 Department of Veterans Affairs (VA) Health Services Research and Development (HSR&D) Expanding expertise Through E-health Network Development (EXTEND) Quality Enhancement Research Initiative (QUERI), Indianapolis, IN USA; 4 VA HSR&D Elizabeth Dole Center of Excellence for Veteran and Caregiver Research, South Texas Veterans Health Care System, San Antonio, TX USA; 5 Department of Medicine, University of Texas Health San Antonio, San Antonio, TX USA; 6 VA HSR&D Center for Health Information and Communication (CHIC), Indianapolis, IN USA; 7 Richard L. Roudebush VA Medical Center, Indianapolis, IN USA; 8 Regenstrief Institute, Indianapolis, IN USA; 9 Department of Biostatistics, College of Public Health, University of Nebraska Medical Center, Omaha, NE USA; 10 Department of Neurology, Indiana University School of Medicine, Indianapolis, IN USA; 11 Department of Biostatistics, Indiana University School of Medicine, IUPUI, Indianapolis, IN USA

**Keywords:** geriatrics, care coordination, program evaluation.

## Abstract

**Background:**

In response to the aging population, the Department of Veterans Affairs (VA) seeks to expand access to evidence-based practices which support community-dwelling older persons such as the Geriatric Resources for Assessment and Care of Elders (GRACE) program. GRACE is a multidisciplinary care model which provides home-based geriatric evaluation and management for older Veterans residing within a 20-mile drive radius from the hospital. We sought to expand the geographic reach of VA-GRACE by developing a hybrid-virtual home visit (TeleGRACE).

**Objectives:**

The objectives were to: (1) describe challenges encountered and solutions implemented during the iterative, pre-implementation program development process; and (2) illustrate potential successes of the program with two case examples.

**Design:**

Quality improvement project with longitudinal qualitative data collection.

**Program Description:**

The hybrid-virtual home visit involved a telehealth technician travelling to patients’ homes and connecting virtually to VA-GRACE team members who participated remotely.

**Approach & Participants:**

We collected multiple data streams throughout program development: TeleGRACE staff periodic reflections, fieldnotes, and team meeting notes; and VA-GRACE team member interviews.

**Key Results:**

The five program domains that required attention and problem-solving were: telehealth connectivity and equipment, virtual physical examination, protocols and procedures, staff training, and team integration. For each domain, we describe several challenges and solutions. An example from the virtual physical examination domain: several iterations were required to identify the combination of telehealth stethoscope with dedicated headphones that allowed remote nurse practitioners to hear heart and lung sounds. The two cases illustrate how this hybrid-virtual home visit model provided care for patients who would not otherwise have received timely healthcare services.

**Conclusions:**

These results provide a blueprint to translate an in-person home-based geriatrics program into a hybrid-virtual model and support the feasibility of using hybrid-virtual home visits to expand access to comprehensive geriatric evaluation and ongoing care for high-risk, community-dwelling older persons who reside geographically distant from the primary VA facility.

**Graphical Abstract:**

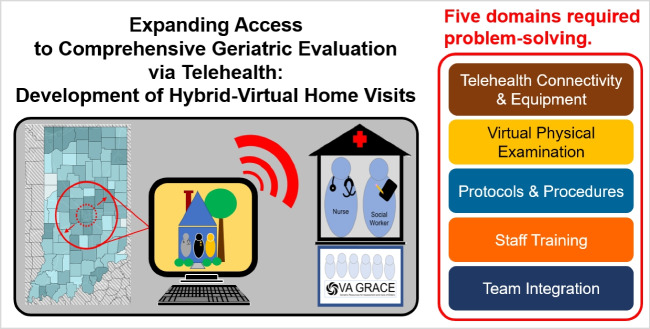

**Supplementary Information:**

The online version contains supplementary material available at 10.1007/s11606-023-08460-5.

## INTRODUCTION

The Veterans Health Administration (VA) is the largest integrated healthcare system in the United States. The 1999 Veterans Millennium Benefits and Healthcare Act (commonly known as the Mill Bill) requires the VA to provide access to geriatric evaluation.^1, 2^ The demand for comprehensive geriatric care within the VA will continue to increase as the Veteran population ages; one-third of the Veteran population will be ≥75-years-old by 2028.^3^ In response to the aging of the population, the VA healthcare system seeks to expand access to evidence-based models of geriatric care which support community-dwelling older persons.

The Geriatric Resources for Assessment and Care of Elders (GRACE) program is an evidence-based intervention targeting high-risk, community dwelling older adults. GRACE is a collaborative, multidisciplinary care model providing home-based geriatric management.^4^ VA-GRACE^5^ has been associated with 7.1% fewer Emergency Department (ED) visits, 14.8% fewer 30-day readmissions, 37.9% fewer hospital admissions, and 28.5% fewer bed-days of care, saving the VA hospital an estimated $200,000/year after program costs.^6^

The primary barrier to expanding reach of VA-GRACE is the drive-time for staff making home visits which are an essential component of VA-GRACE.^4^ A meta-analysis of 15 studies of home visits for community-dwelling older persons identified reductions in mortality (odds ratio 0.76; 95%CI 0.64-0.89) and admissions to long-term care facilities (odds ratio 0.65; 95%CI 0.46-0.91) for patients receiving home visits.^7^ Home visits—compared with in-clinic assessments—have been shown to identify new problems and result in new recommendations.^8^ During the COVID-19 pandemic, video visits were adopted in lieu of in-home visits for geriatrics patients enrolled in home based primary care (HBPC).^9, 10^ Among HBPC patients who had not previously used telehealth, 27% were unable to use video visits and 82% required assistance to complete the video visit.^10^ Therefore, our challenge was to develop an approach that expanded access to home visits for community-dwelling older persons living beyond the constraints of the 20-mile drive distance, and overcame the reported barriers to geriatric telehealthcare.^10–12^

The objective of this quality improvement (QI) project was to expand the geographic reach of the VA-GRACE program by developing a hybrid-virtual home visit to create a telehealth version of VA-GRACE (referred to as TeleGRACE). The purpose of this manuscript was to: (1) describe challenges encountered and solutions implemented during the iterative program development process; and (2) illustrate potential successes of TeleGRACE using two case examples. This manuscript adheres to SQUIRE 2.0 guidelines.^13^

## METHODS

### Context: VA Geriatrics

The VA offers a broad range of geriatric programs. The VA Geriatric Research, Education, and Clinical Centers (GRECC) Connect program provides education and clinical support for providers caring for rural-dwelling older Veterans. The VA geriatric clinical programs span hospital, outpatient, and long-term care settings. The VA has implemented a variety of community-based geriatric programs serving older adults with complex health and social needs including Home Based Primary Care (HBPC), Medical Foster Home, VA Caregiver Support Program, Resources Enhancing Alzheimer’s Caregiver Health (REACH), Caregivers of Older Adults Cared for at Home (COACH), and Veteran Directed Care.^14^

Although many VA geriatrics clinical programs involve multidisciplinary teams, VA-GRACE differs from HBPC and geriatric patient aligned care teams (Geri-PACT) because VA-GRACE patients continue to receive primary care services from their VA PACT teams. In many cases, VA-GRACE patients have long-standing relationships with their PACT providers.^14^ The VA-GRACE team collaborates with primary care in a co-management model to optimize and implement patient-centered care plans. VA-GRACE supports primary care staff by offering geriatrics clinical expertise for patients who may have geriatric syndromes but do not require intensive geriatric care.

VA-GRACE also differs from the hospital-in-home model which delivers short-term, acute hospital-level care for medically stable patients within the home setting.^15^ Although, VA-GRACE staff make home visits after ED or inpatient hospital stays, VA-GRACE is not focused on acute medical conditions, but rather provides longitudinal, multidisciplinary care in the outpatient setting.

### Setting & Patient Participants

This QI project was conducted at the Richard L. Roudebush VA hospital (October 2020-September 2021: pre-implementation iterative program development; October 2021-April 2022: pilot-implementation; May 2022-September 2024: active implementation). This manuscript describes the iterative program development and pilot-implementation phase.

The eligibility criteria to receive VA-GRACE was: age ≥65 years-old, residing at home [not rehabilitation or skilled nursing facilities], residence ≤20 miles of the facility, not receiving hospice care, not on dialysis, not receiving HBPC, had a VA primary care visit within the prior 2 years, and no active substance abuse. Our goal for TeleGRACE was to provide access to comprehensive geriatric services to high-risk, community-dwelling older Veterans. We focused on Veterans who were being discharged from a VA inpatient admission or ED visit. Exclusion criteria for TeleGRACE were: age <70-years; already enrolled in VA-GRACE, HBPC, geriatric primary care, or the complex transitions of care program; residence >60 miles from the facility; no geriatric syndrome; and Care Assessment Need [CAN] score <95 percentile (patients who otherwise met inclusion criteria but did not have a CAN score were not excluded). The CAN score predicts risk of hospitalization or death.^16, 17^ TeleGRACE received referrals from the inpatient geriatrics service, inpatient admitting teams, ED, and primary care. The TeleGRACE team also examined the facility’s ED and inpatient discharge lists (via an electronic dashboard) to identify potentially eligible patients. Manual chart review was conducted to ensure potentially eligible patients had a geriatric syndrome. All eligible patients were called and offered enrollment.

### VA-GRACE Program

The VA-GRACE team includes dyads of nurse practitioners paired with social workers who conduct home visits. Home visits include screening for common geriatric syndromes, focused physical examination, medication reconciliation, and psychosocial assessment. The dyads present cases to the VA-GRACE multidisciplinary team during weekly team rounds facilitated by a geriatrician (CCS) and including a psychologist and pharmacist. A multidisciplinary care plan is developed during the team rounds and shared via the electronic medical record with patients’ primary care providers. Typically, VA-GRACE team members place orders for durable medical equipment (e.g., walkers, grab bars) but collaborate with primary care providers on medication management.

The VA-GRACE dyad returns to patients’ homes and ensure patients and caregivers understand—and agree with—each element of the care plan. Ongoing communications between patients and VA-GRACE dyads take place monthly and as-needed, generally via telephone. Follow-up assessments (by telephone or in-person) are made after any ED visit or admission. Home visits are conducted annually to assess changes in condition or social supports. Patients remain enrolled in VA-GRACE until functional and clinical improvement such that VA-GRACE support is no longer needed, long-term care placement, patients or caregivers request discharge, persistent nonadherence to VA-GRACE recommendations, or death.

### Quality Improvement Intervention: Development of a Hybrid-Virtual Home Visit

The only modification to VA-GRACE made for the TeleGRACE QI project was the development of a hybrid-virtual home visit^18–21^ to enroll patients living ≤60 miles from the hospital. Prior studies of “virtual home visits,” using either telephone or video visits to connect patients’ homes with providers, identified barriers to implementation including: lack of internet connectivity and other technology access issues, sensory (visual and hearing) and cognitive impairments, reliance on caregivers, addressing sensitive topics, and incomplete examinations.^10–12^ Based on experiences of VA-GRACE team members and existing literature, we developed a hybrid-virtual home visit model where a telehealth technician travelled to patients’ homes and connected virtually to VA-GRACE team members who participated remotely.

### Iterative Program Development Process

TeleGRACE team members—including the geriatrician leader of the VA-GRACE program (CCS), a primary care provider and health services investigator (DMB), project coordinator (ALS/JM), and telehealth technician (SF)—met twice-weekly to reflect on our experiences, identify gaps in the program or opportunities for enhancement, and make plans for pilot-testing innovations. Members of the TeleGRACE team connected with VA-GRACE staff regularly to identify emergent issues and monitor progress. The VA-GRACE program convenes a monthly quality improvement session where TeleGRACE was discussed with all team members.

### Conceptual Frameworks

We used the RE-AIM^22, 23^ framework and the Consolidated Framework for Implementation Research (CFIR)^24^ to guide the QI approach and evaluation. We used both deductive (based on the frameworks) and inductive (emerging from the data) coding in the rapid qualitative analysis.^25, 26^

### Data Collection

We collected multiple data streams throughout program development: periodic reflections with staff designing and implementing TeleGRACE; fieldnotes from staff implementing hybrid-virtual home visits; TeleGRACE team meeting notes; and VA-GRACE team member interviews.

The periodic reflections were collected using a guided discussion template (Appendix A) designed to systematically document pre-implementation and pilot-implementation activities including challenges, facilitators, networks and communications, leadership engagement, and use of data to make protocol adaptations.^27^ The template was pre-populated with deductive concepts related to both the RE-AIM (e.g., implementation barriers) and CFIR (e.g., goals and feedback) frameworks. Thirty-minute individual, guided interviews using Microsoft TEAMS were conducted quarterly by the evaluation team (TMD, AP) with staff designing and implementing TeleGRACE (CCS, SF, ALS, JM, DMB). Reflection notes were taken during the sessions and summarized in the template. Each quarter, the evaluation team reviewed all periodic reflections and synthesized main themes concerning implementation activities, barriers, and adaptations.

The telehealth technician was trained by a master’s-level anthropologist in journaling methods about general experiences and specific patient interactions. The evaluation team reviewed journal entries and provided feedback to ensure the document captured the telehealth technician’s lived experience, areas for improvement, and reflections on how innovations were either working or not working from their perspective. The journal entries were reviewed by the evaluation team for themes.

## RESULTS

### Hybrid-Virtual Home Visit Intervention Development: Challenges and Solutions

**Figure 1:**
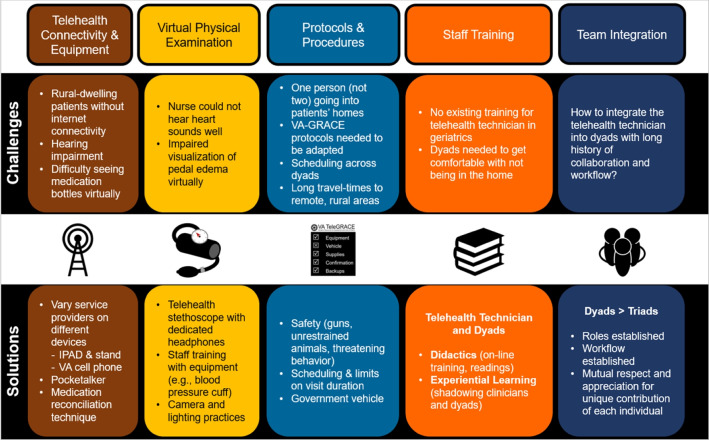
Challenges encountered and solutions developed during the iterative construction of the hybrid-virtual home visits. Legend. The challenges that were encountered during the development of the TeleGRACE program fell within six domains: telehealth connectivity and equipment issues, virtual implementation of the physical examination, the need to develop new protocols and procedures, staff training, identification of eligible patients, and team integration. The figure identifies examples of these challenges and solutions that were developed during the iterative process that was used to design the approach to hybrid-virtual home visits.

Challenges encountered during development of the hybrid-virtual home visit fell into five domains: telehealth connectivity and equipment, virtual physical examination, protocols and procedures, staff training, and team integration (Fig. 1). Through a process of iterative program development, solutions were designed and pilot-tested for each problem.

#### Telehealth Connectivity & Equipment

Many TeleGRACE patients lived in rural areas with poor cellular and internet connectivity. The telehealth technician had multiple devices (e.g., IPAD, laptop, VA cell phone) that could connect with the remote VA-GRACE dyads; each device used a different cellular carrier. Hearing impairment was a barrier to communication between patients and team members connecting remotely. VA-provided “Pocketalker” personal amplifiers facilitated communication, but in some cases of severe hearing loss, the telehealth technician served as a communication-intermediary between patients and remote staff. A critical element of VA-GRACE is medication reconciliation. By iteratively modifying camera angles, the telehealth technician implemented virtual medication reconciliation using the IPAD with a stand to show remote staff patients’ medication bottles and pill boxes.

#### Virtual Physical Examination

VA-GRACE nurse practitioners conduct physical assessments with patients during home visits. It was imperative the nurse practitioners were satisfied that virtual physical examinations provided information needed for clinical decision-making. The telehealth technician was trained in physical examination during in-person clinic visits in primary care and in-person home visits with each of the three VA-GRACE dyads, including: vital signs measurement (e.g., use of a blood pressure monitor, scale, oximeter); telehealth-enabled stethoscope use; and instructing patients in breathing maneuvers. Several iterations were required to identify the combination of telehealth stethoscope with dedicated headphones that allowed remote nurse practitioners to hear heart and lung sounds. The telehealth technician modified camera angles and lighting so remote clinicians could appreciate edema severity. The physical examination element not virtually adapted was the ear exam; acquisition of telehealth otoscopes was delayed by contracting issues (between VA and telehealth vendors).

#### Protocols & Procedures

**Figure 2:**
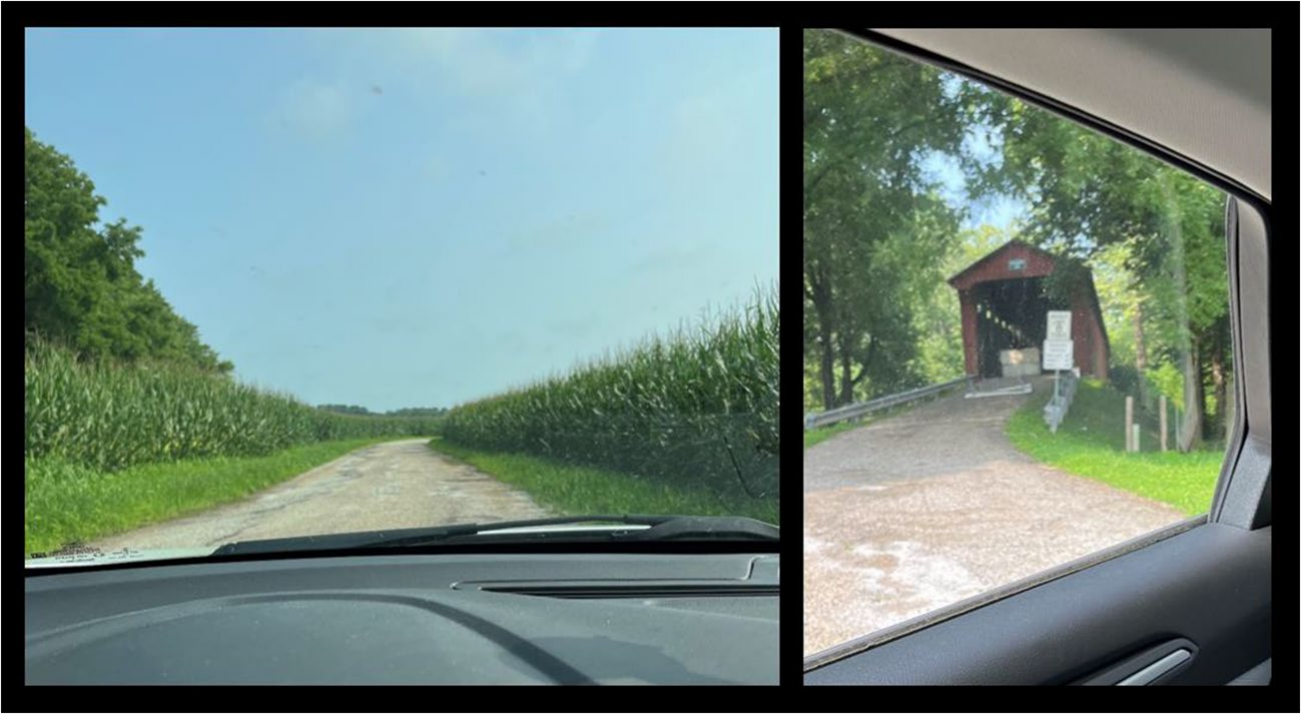
Challenges of providing in-home services in rural settings. Legend. Although many of the TeleGRACE patients live in urban settings, the photograph on the left illustrates that the telehealth technician sometimes travels through rural regions without ready access to safety services. The photograph on the right illustrates the situation where mapping services direct the telehealth technician to use a roads that are closed (e.g., due to contruction or in this case due to a bridge that was in disrepair).

The VA-GRACE program’s operating manual was designed for two individuals making in-person home visits. Safety concerns were amplified with only one person in patients’ homes (e.g., Fig. 2 illustrates challenges with the rural setting). Existing VA-GRACE policies applied to TeleGRACE included: patient or caregiver threatening behavior; home setting safety concerns (e.g., unsecured guns, unrestrained animals); program adaptations during staff shortages and for COVID-19 positive patients; and regulations regarding mileage reimbursement and use of government vehicles. New TeleGRACE policies were developed for hybrid-virtual home visit scheduling considering travel logistics (i.e., time to drive from one patient home to the next) and visit duration (e.g., split assessment into two visits if encounters exceeded 90-minutes). When scheduling visits, the telehealth technician had to be provided opportunities for breaks (e.g., to use public restrooms). Given the time spent driving to patients’ homes, it was essential the telehealth technician not attempt visits when patient were not at home (no-shows); hence, new procedures were implemented to contact patients 3-days prior to and on the day of the scheduled visit.

VA-GRACE protocols specify if any staff member (including the telehealth technician) feels unsafe, they should terminate the home visit. The entire team—but especially the telehealth technician—was encouraged to voice any safety concerns they had for themselves or patients.

#### Staff Training

The telehealth technician had to be trained on diverse topics for which no single resource existed: geriatrics in general, and VA-GRACE assessments (e.g., cognition assessment, depression screening); telehealth; physical examination; cardiopulmonary resuscitation (CPR); mental health and suicidality; and government vehicle policies. Training was conducted using online resources (Appendix B) as well as experiential, in-person learning activities. VA-GRACE nurse practitioners had to be trained to use virtual stethoscopes. VA-GRACE dyads had to become comfortable not being in patients’ homes, which required time working with the technology and telehealth technician. A key element of staff training was having the telehealth technician accompany the three VA-GRACE dyads on two of their in-person visits until all team members were satisfied with the procedures the telehealth technician used to conduct the hybrid-virtual home visit.

#### Team Integration

The VA-GRACE team members were committed to expanding access to VA-GRACE; it was a shared belief that VA-GRACE provides highest quality of care and the desire reach more Veterans that motivated collaboration to build a hybrid-virtual home visit program. VA-GRACE team members initially worried that substituting hybrid-virtual home visits for in-person home visits would degrade interactions with patients and caregivers and result in lower quality of care. The primary areas of concern were the ability to conduct medication reconciliation virtually, interacting with patients who are in distress remotely, having confidence in a virtual physical examination, and trusting the equipment would function without time-intensive troubleshooting.

The VA-GRACE program has been implemented since 2010. The integration of the new telehealth technician into existing VA-GRACE team dyads was an implementation challenge. Transitioning from dyads to triads required: establishment of roles and responsibilities; workflow management; balancing caseloads, especially during intermittent staff shortages; and development of mutual respect and appreciation for the unique contribution of each individual. Changing the team’s language from “dyads” to “triads” was a key step in this evolution. In addition, the geriatrician-leader of VA-GRACE emphasized that the telehealth technician was part of the clinical team and not merely providing administrative support. During weekly multidisciplinary team meetings, the telehealth technician started every patient report with their own observations. The triads’ direct experience of successful home visits promoted confidence in the TeleGrace delivery model (i.e., being “pleasantly surprised” by the program’s success).

### Clinical Case Examples

Case 1: A patient who had been scheduled for an initial TeleGRACE enrollment visit after being discharged from a VA admission, sought care for a lower extremity wound in a non-VA ED closer to his home than the VA facility. During the TeleGRACE visit (scheduled 12 days after the non-VA ED visit), the telehealth technician conducted a physical assessment with the VA-GRACE nurse practitioner participating remotely. The nurse practitioner saw the wound and discovered the patient had not received wound-related follow-up care. Neither wound care supplies nor home health care had been provided at discharge from the non-VA ED visit. During the TeleGRACE hybrid-virtual home visit, the telehealth technician sent pictures of the wound to the VA-GRACE nurse practitioner who placed an electronic consult to the VA wound care service. The wound care team reviewed the pictures, determined the appropriate plan of care, collaborated with the VA-GRACE social worker to order home health wound care, and sent wound care supplies to the patient’s home, all during the TeleGRACE visit. The patient lived >45 miles from the VA medical center and would not have qualified for in-person VA-GRACE; this single TeleGRACE visit altered the typical trajectory of care, which would likely have included an in-person visit in primary care and a separate visit in the wound care clinic.

Case 2: During a TeleGRACE enrollment visit, a patient who had been discharged from a VA inpatient stay 13 days previously became weak and unwell. The telehealth technician obtained vital signs with the nurse practitioner participating remotely; the patient’s oxygen saturation was 84% and heart rate was 178 beats/minute. The nurse practitioner recommended the patient seek ED care. The telehealth technician volunteered to call 911, but the wife declined and drove the patient to the ED. The nurse practitioner called the VA ED to discuss the case with the medical officer on duty. When the patient arrived at the ED, he was fasted-tracked through triage and ultimately admitted to the hospital for a 5-day stay. The patient told the ED staff and inpatient teams that TeleGRACE saved his life.

## DISCUSSION

Prior studies have identified many barriers to implementing virtual home visits for geriatric populations.^10–12^ Our results demonstrate the feasibility of developing and implementing a hybrid-virtual home visit model to care for high-risk, community-dwelling older persons. The cases illustrate how this model cared for patients who might not otherwise have received timely healthcare.

The five domains of pre-implementation activities requiring problem-solving were: telehealth connectivity and equipment, virtual physical examination, protocols and procedures, staff training, and team integration. We anticipated some challenges (e.g., limited internet availability, patients’ hearing impairment), but some challenges were unexpected. For example, the telehealth technician was allergic to cats; yet, many patients had cats in the home. Visitors in the home can be distracting during in-person home visits but can disrupt hybrid-virtual home visits when staff participating virtually have trouble hearing the patient, caregiver, or telehealth technician. Higher gas prices made mileage reimbursement for personal vehicle use less affordable; however, the process of reserving, obtaining, using, and returning government vehicles is complex and time-intensive. Although these have not be unsurmountable difficulties, the challenges we described should be considered when implementing hybrid-virtual home visits. The literature suggests that staff education via simulations,^28^ checklists,^29^ use of electronic health record systems to facilitate communication,^21^ and caseload management^30^ may be useful when implementing home visit programs.

Hybrid-virtual models of care involving a support person in the home with a remote clinical team have been evaluated as an approach to providing advanced care in home settings (“hospital at home”).^18–20^ In those models, nurses or community paramedics visited patients’ homes which were monitored remotely by command center clinical staff . Those hybrid-virtual programs demonstrated high patient satisfaction.^19^ Although VA geriatrics programs used telephone or video visits in lieu of in-person home visits during the COVID-19 pandemic, we are unaware of other VA geriatric programs implementing the hybrid-virtual home visit model. Future research is needed to understand whether existing healthcare infrastructure (e.g., home healthcare agencies) can fill the role of the telehealth technician to facilitate communication between patients and virtual providers.

Several limitations merit description. First, this hybrid-virtual home visit model was implemented at the only VA site currently implementing GRACE; results may vary at other hospitals and in other geographic regions. For example, although rarely encountered, we did not record the number of times a visit had to be modified due to internet connectivity problems which may vary by rurality. Second, the program was implemented with the VA. Future research should evaluate the effectiveness of hybrid-virtual home visits implemented in non-VA sites^31^ where the healthcare infrastructure (e.g., electronic health record; availability of clinical pharmacists and health psychologists) may vary. Third, the data collected to inform iterative program development were principally collected from VA staff. Although we sought feedback from patients about the hybrid-virtual home visits, patients and caregivers were not formally interviewed. An assessment of TeleGRACE is currently underway; patients and caregivers are being interviewed as part of that evaluation. Moreover, this manuscript focused only on the development and initial pilot-testing of TeleGRACE; details about the number of eligible patients who agreed to participate and program effectiveness will be reported in a future manuscript describing the evaluation of the active implementation of TeleGRACE. Fourth, this program was developed and pilot-tested during the COVID-19 pandemic when preferences about having visitors in one’s home were changing compared with the pre-pandemic period. Future research should examine how home visit preferences change over time. Finally, this pilot program enrolled eligible Veterans after VA inpatient stays or ED visits because of the inability to access information about non-VA healthcare in a timely fashion; future studies should examine how to leverage data from the VA health information exchange and community care programs to expand the reach of VA geriatric services to high-risk older Veterans discharged from non-VA care settings.^32, 33^

### Conclusions

The TeleGRACE program has expanded access to comprehensive geriatric assessments and ongoing care management to high-risk, community-dwelling older persons whose reside geographically distant from the primary VA medical center. Widespread deployment of programs like the TeleGRACE hybrid-virtual home visit model will be required to support the Veteran population as they age in place.

### Supplementary Information

Below is the link to the electronic supplementary material.

Supplementary file1 (DOCX 32 KB)

## Declarations:

### Data Sharing Statement

The datasets used during the current study contain personal health information and are therefore not publicly available.
