# Supplementary material for: Expanding Access to Comprehensive Geriatric Evaluation via Telehealth: Development of Hybrid-Virtual Home Visits
Source: J Gen Intern Med. 2024 Jan 16;39(Suppl 1):36–43. doi: 10.1007/s11606-023-08460-5 (PMC10937878; doi:10.1007/s11606-023-08460-5)
Supplement: Supplementary file 1 — Supplementary file1 (DOCX 32 KB) [file 11606_2023_8460_MOESM1_ESM.docx]

**Appendix A. Periodic Reflection Interview Guide**

Project member(s):

Date:

Interviewer and note taker:

**Headnotes**

(Quick overview of the event or interaction – anything that happened prior to conducting the periodic reflection that would be useful context for interpreting the reflection notes)

**Detailed Notes**

(Document any additional questions you ask that are not on the script)

These periodic reflections are an opportunity for us to regularly check in about how things are going with the implementation of TeleGRACE. Our main goal is to take a few minutes to reflect on and document key activities, events, and changes that have recently occurred. To try to avoid duplication of data collection efforts, we will focus on implementation at the overall project level, as well as your insights into what is happening in implementation efforts at the site level which may not be captured elsewhere.

1. What have been the overall project’s main implementation activities over the past month/couple months? (probes: recruitment, implementation strategies)

Briefly, what have been the main activities for the implementation site?

How are things going? (probes: progress toward goals)

1. What, if any, sources of data are you looking at to understand how implementation is going?
2. Have there been any changes to how the intervention is delivered in the past month? (probe: what prompted the change)
3. Have there been any changes to the implementation plan in the past month? (probes: what prompted the change, adaptations to implementation strategies, expected impacts, changes to goals)
4. Who have been the key people involved in recent activities, efforts, and discussions? (probes: who is working together/coordinating, relationships)
5. Have any barriers or concerns recently arisen? How have those been addressed?
6. For project PIs only: Have there been any recent efforts to engage stakeholders?

(probes: informally vs formally, project-specific partners)

1. Have you seen any recent changes in the local or national environment that you think may have impact for implementation?
2. What lessons have been learned?
3. What are the next steps going forward?

**Appendix B. Telehealth Technician Training Checklist**

**TeleGRACE Required Talent Management System (TMS) Modules**

- HeartCode® BLS Complete (VA 3871645)
- PMDB Training Videos: PMDB Geriatric and Dementia Module (VA 41018)
- Abuse and Neglect of Older Adults (NFED 99904)
- Physical and Mental Assessment of the Older Adult (VA 35745)
- Dementia Care: Understanding Alzheimer's Disease (NFED 4628479)
- Assessment: Orthostatic Vital Signs – CE (NFED 100389)
- Assessment: Pressure Injury and Wound – CE (NFED 99966)
- Cultural Considerations for Conducting Trauma-Focused Treatment with Latinx Patients (VA 131001457)
- Hand Hygiene – CE (NFED 100201)
- Introduction to Connected Care (VA 4486691)
- Introduction to Telepresenting (VA 4460923)
- Measuring Vital Signs (NFED 4628531)
- Medical Record Documentation & Legal Information for Certified Nursing Assistants (NFED 4628709)
- PMDB Training Videos: PMDB Medical Management Module (VA 41019)
- The Role of the TCT (VA 4492400)
- SLUMS (VA 39921)
- Introduction to System Based Telepresenting (VA 4486003)
- Telewound: The Patient Encounter (VA 4450924)
- Cultural Communication/Awareness (VA 1717667)

**VA-GRACE Required TMS Modules**

- Defensive Driving 2.0 (NFED 4503953)
- Emergency Preparedness, Fire Safety, and Hazardous Materials (VA 12438)
- GEMS General Awareness Training (VA 935)
- General Compliance Awareness Training for Revenue Staff (VA 44203)
- Government Ethics - The Essentials (VA 3812493)
- Harassment Prevention & Accountability Training (VA 45224)
- HRO Baseline Curriculum Program - HRO Baseline Training for Staff Members (VA 40879)
- Infection Control: Bloodborne Pathogens and Tuberculosis (VA 12434)
- Mobile Training: Security of Apps on iOS Devices (WBT) (VA 3926744)
- Own the Moment (VA 43391)
- Patient Safety and Reporting Patient Incidents (VA 4686)
- Privacy and HIPAA Training (VA 10203)
- Personal Protective Equipment (PPE) Guidance (VA 4554216)
- Population-Specific Care and Cultural Differences (VA 12414)
- Preventable Healthcare Associated Infections (HAI's) (VA 1291953)
- Prevention and Management of Disruptive Behavior (PMDB) (VA 131001568)
- Relationships in the Workplace (VA 4278984)
- Suicide Prevention Guide Training (S.A.V.E.) (VA 66979)
- The EEO, D&I, No FEAR, and Whistleblower Rights and Protection Policy Statement (VA 430985)
- The Notification and Federal Employee Antidiscrimination and Retaliation (No FEAR) Act Training (VA 45316)
- Unauthorized Commitments (UAC) (VA 1701572)
- Utilities Management Equipment Management Electrical Safety Sticker Accident Reporting (VA 12435)
- V10 Patient's Bill of Rights and Identifying Victims of Abuse (VA 12172)
- VA Core Values Training (I CARE) (VA 3901227)
- VA Fleet Card Training (VA 5872)
- VHA Driver Safety - Accessible Version (VA 7350)
- VA Privacy and Information Security Awareness and Rules of Behavior (WBT) (VA 10176)
- VA Video Connect e911 Training for Non-Clinical Telehealth Staff (VA 4626496)
- VA Video Connect to Home (VVC): Integrated Training (VA 4556649)
- VHA Journey to High Reliability - HRO 101 (VA 37667)
- Whistleblower Rights and Protections (VA 39953)
